# A Network Meta-Analysis of the Clinical Efficacy and Safety of Commonly Used Chinese Patent Medicines in the Auxiliary Treatment of Poststroke Depression

**DOI:** 10.1155/2022/7265769

**Published:** 2022-01-06

**Authors:** Ying Yu, Gong Zhang, Tao Han, Hongjie Liu, Hailiang Huang

**Affiliations:** ^1^Innovative Institute of Chinese Medicine and Pharmacy, Shandong University of Traditional Chinese Medicine, Jinan 250355, China; ^2^College of Integrated Traditional Chinese and Western Medicine, Shandong Liming Vocational College of Science and Technology, Tai'an 271000, China; ^3^Graduate Office of Shandong University of Traditional Chinese Medicine, Jinan 250355, China; ^4^School of Traditional Chinese Medicine, Jinan University, Guangzhou 510632, China; ^5^College of Rehabilitation Medicine, Shandong University of Traditional Chinese Medicine, Jinan 250355, China

## Abstract

**Background:**

Poststroke depression (PSD) is a serious complication of clinical cerebrovascular disease. Patients not only have depression-related emotional symptoms but also have physical symptoms, such as autonomic dysfunction. At the same time, patients with varying degrees of depression will delay the neurological function of stroke patients. The recovery time of cognitive function and limb function will increase the risk of accidental death and even aggravate the mortality of cerebrovascular disease. Through combining data analysis and related literature, seven types of Chinese patent medicines (CPMs) widely used in the clinical treatment of PSD have been screened out. These herbs exhibit some clinical comparability under the conditions that the syndrome type and dosage form are relatively uniform. Therefore, in this study, the network meta-analysis method was used to evaluate the safety and efficacy of the seven CPMs screened out, and the probability ranking was performed to screen the best clinical auxiliary treatment plan of CPM.

**Methods:**

We searched the Chinese databases, including CNKI, WANFANG, and VIP, as well as the English databases, including the Cochrane Library, EMBASE, and PubMed, from inception to May 31, 2020, to identify randomized controlled trials (RCTs) on seven kinds of CPMs that were the subjects of the clinical research. The bias risk and quality of the included studies were analyzed with the Cochrane Handbook (version 5.1), ADDIS, and R software, and the results were compared in a network meta-analysis (NMA).

**Results:**

In terms of clinical effectiveness, the seven kinds of CPMs all improved clinical curative effects, with Jieyu Anshen capsule adjuvant treatment having the most significant effect [odds ratio (OR) = 5.00, 95% CI (1.72–9.48)]. Wuling capsule AT can effectively reduce the score index of scale factors for the HAMD score, NIHSS score, and TESS score [mean difference (MD) = −3.95, 95% CI (−4.88–3.00); OR = −3.25, 95% CI (−5.46)–1.05); OR = 0.22, 95% CI (0.05–0.79), resp.].

**Conclusion:**

The mechanisms of seven CPMs in the adjuvant treatment of PSD have advantages. In terms of safety and efficacy, the CPMs had better clinical adjuvant treatment performance. Although this study concluded that the Jieyu Anshen capsule is the preferred drug for clinical treatment, a clear conclusion still needs to be verified in a high-quality randomized controlled study. In clinical practice, accurate selection and application can be carried out according to the specific characteristics of patients.

## 1. Introduction

Poststroke depression (PSD) is a serious complication of cerebrovascular disease, which is frequently observed within three to six months following stroke onset, with an incidence of approximately 22–75% [1]. Patients not only have symptoms related to autonomic nervous system and other physiological distress, but also have depression-associated emotional symptoms. Different degrees of depression can also attenuate the recovery of neurological functions, cognitive functions, and limb functions in these patients. Depression can further increase the risk of accidental death and even aggravate the mortality of cerebrovascular disease [2, 3]. Thus, it is of great importance to improve clinical efficacy, enhance neurological functions, and enhance the quality of life of patients. To date, conventional antidepressant medications are widely used along with western drugs. Although they are effective for increasing monoamine transmitter levels in the synaptic space of neurons, relieving depressive symptoms, and prolonging the treatment duration, patients can experience varying degrees of side effects and may easily relapse after treatment discontinuation. This severely limit patient's adherence to medication and the curative effects following stroke [4, 5].

Traditional Chinese medicine (TCM) has been shown to offer advantages for the treatment of strokes. Recently, Chinese patent medicine (CPM), in combination with western drugs, is commonly applied for treating this disease. CPM not only effectively avoids drug resistance, toxicity, addiction, and other defects that are seen with long-term application of western drugs but also mediates the visceral functions. CPM can rapidly relieve depression, reduce rehabilitation times, and enhance the quality of life. According to previous literature, seven types of oral CPMs widely used for the treatment of PSD were screened out. These are considered to be most representative, and they have some clinical comparability under the conditions such that the syndrome types and dosage forms are relatively uniform. However, the majority of meta-analyses have only described the efficacy of oral CPM in the treatment of PSD, and no evidence-based assessment has been conducted on the safety and efficacy of the seven representative CPMs in treating PSD. Hence, this network meta-analysis (NMA) aimed to provide the clinically relevant evidence of direct and indirect comparisons and comprehensively analyze the efficacy and safety of CPMs in the treatment of PSD. Additionally, the most ideal therapeutic approach was chosen to facilitate evidence-based clinical decisions for the optimization of combinatorial drug therapies.

## 2. Materials and Methods

### 2.1. Information Sources

Using computer retrieval technology, we searched for clinical randomized controlled trials (RCTs) of seven types of oral CPMs for the adjuvant treatment (AT) of PSD [6]. Primary searching was conducted from the establishment of the database to 31 May 2020. We searched Chinese databases, including CNKI, WANFANG, CBM, and VIP, as well as English databases, including the Cochrane Library, EMBASE, Web of Science and PubMed. The key terms included CPM, Wuling capsule, Shugan Jieyu capsule, Yangxue Qingnao granule, Jieyu Anshen capsule, Chaihu Shugan powder, Danzhi Xiaoyao pills, Xiaoyao pills, depression syndrome after stroke, PSD, and RCT. Different combinations of keywords, free words, and subject words were chosen for different databases.

During literature searching, the free words and subject words were independently searched, and the relevant keywords were employed for comprehensive searches. In addition, potential trial registrations were searched through the ClinicalTrials.gov and WHO international clinical trials registration platform. The references in the relevant journals were searched and tracked. Different search engines, such as Baidu academic, Google scholar and others, were used for manual searches. Data from major researchers and relevant authors were included to supplement incomplete reports or unpublished data from the original articles, in order to ensure comprehensive primary searches. In accordance with the Participant-Intervention-Comparator-Outcomes-Study (PICOS) principles, the studies that met the standards were included.

### 2.2. Eligibility Criteria

The selection criteria of this NMA were in accordance with the five main principles of PICOS.

#### 2.2.1. Characteristics of Participants

The participants were patients with PSD and their inclusion was not limited by age, gender, or race. The diagnostic standard for PSD was based on the “Guidelines for the Diagnosis and Treatment of Acute Ischemic Stroke in China” (2014 Edition) revised by the Cerebrovascular Diseases Group of the Neurological Branch of the Chinese Medical Association in 2014, and stroke was in the sequelae period or recovery period [7, 8]. The diagnostic standard for depression was based on the “Classification and Diagnosis Criteria of Chinese Mental Disorders” (3rd Edition) [9]. The efficacy of TCM was assessed based on the diagnostic standards for stroke and depression [10].

#### 2.2.2. Intervention and Comparator Types

When the judgment criteria of diagnosis and curative effects are clear and consistent, the experimental group is subjected to CPM treatment (Wuling capsule, Shugan Jieyu capsule, Yangxue Qingnao granule, Jieyu Anshen capsule, Chaihu Shugan powder, Danzhi Xiaoyao pill, or Xiaoyao pill) combined with western drugs, while the control group is subjected to western drug administration.

#### 2.2.3. Types of Outcomes

Outcomes were described as (1) HAMD score, (2) TESS score, (3) NIHSS score, and (4) clinical efficacy.

#### 2.2.4. Type of Study

All of the included studies were RCTs with blind or assignment concealment and no language limits.

### 2.3. Exclusion Criteria

We excluded nonrandomized controlled trials, case reports, experience summaries, self-controlled studies, review articles, animal studies, and repeated publications. The diagnosis of PSD is not always clear and studies sometimes combine stroke with other diseases. The efficacy judgment standard for the control and experimental groups was not always clear, and the treatment measures involved other treatments that could affect the final outcome of the causality judgment literature. Studies with incomplete data and unclear research findings or no connection with the full-text authors were also excluded.

### 2.4. Literature Screening

**Figure 1:**
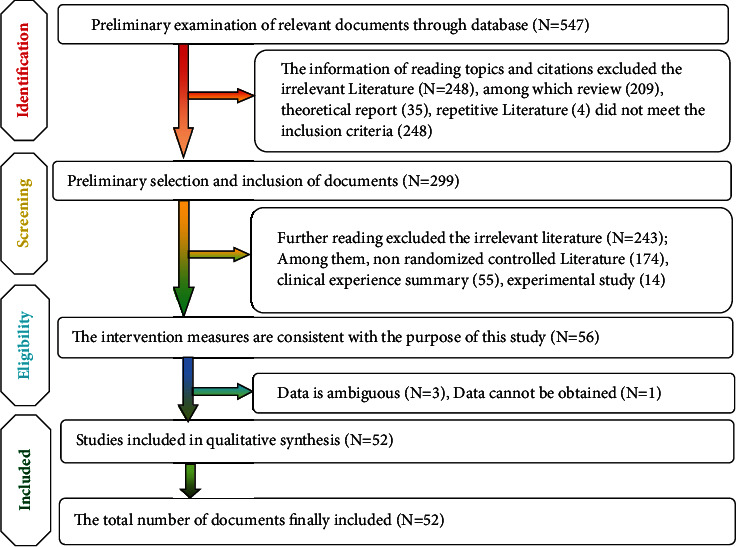
A flowchart of literature screening.

The electronic databases were searched by three researchers, and EndnoteX9 software was used to search the redundant information. The studies were combined when retrieval results were found in different databases. All data in the databases were retrieved and the full texts were downloaded. Two independent researchers conducted data extraction according to the preformulated method and the results were cross-checked and reviewed.  Figure 1 shows the PRISMA flowchart describing the process of study selection [11, 12].

### 2.5. Data Extraction

The following data were extracted: (i) baseline information of the included RCTs, such as the first author, year, research topic, published journal, and so on; (ii) relevant information on the control and treatment groups in this study, such as the number of cases, age, treatment course, intervention measures, and outcome indicators; (iii) design types and quality evaluations of the included RCTs; (iv) outcome measures such as HAMD, TESS, NIHSS scores, and clinical efficacy.

### 2.6. Quality Evaluation

The quality of each RCT was evaluated by RevMan based on the Cochrane manual, including assignment concealment, random method, outcome data integrity, blind method, number of dropped cases, selective report, follow-up, and other biases, which was categorized into three groups: uncertainty risk, low risk, and high risk. The two first authors independently completed the quality evaluations of the included RCTs. If the results showed significant differences, a third researcher was invited to discuss and interpret the results and performed a quality evaluation. Literature quality and risk of bias assessments were also conducted based on the Cochrane manual. R v3.3.1 and ADDIS V1.16.5 were employed for statistical analysis, data integration, and NMA [13–15].

### 2.7. Publication Bias

The publication bias was assessed by R software when >5 RCTs were included. A symmetric inverted funnel shape indicates low or no publication bias. On the contrary, an asymmetrical shape indicates a potential publication bias.

### 2.8. Statistical Analysis

RevMan software was used for the assessments of literature quality and risk of bias. ADDIS and R software were employed for direct and indirect result comparisons and 95% CI calculations in the NMA. Meanwhile, anecdotal sequence and network relationship diagrams of the seven types of oral CPMs were constructed, in order to reveal the indirect comparative relationships among them. The node indicates a CPM type, the line denotes a direct or indirect comparative relationship between 2 CPMs, and the line thickness reflects the number of included RCTs. Subsequently, all direct and indirect comparisons were evaluated to determine the most effective CPM for PSD among these seven types of CPMs and estimate the rank probability of CPMs using the Markov chain Monte Carlo (MCMC) method. The ‘NETMETA' program in R package was used, and the Bayes MCMC algorithm was called to analyze the random effects model results [16].

The odds ratio (OR) and 95% confidence interval (CI) were utilized for safety and efficacy analysis, such as the occurrence of side effects and recovery time of PSD symptoms. All measurement data were presented as standardized and weighted mean differences. According to the NMA probability ranking, the higher the total effective rate (Rank 1), the greater the effects, while the smaller the HAMD, NIHSS, and TESS scores (Rank 1), the greater the effects. The data of random effects model were called by ADDIS software according to the Bayesian MCMC algorithm for prior evaluation and processing (four chains were subjected to simulation modelling, and the initial value, iteration step, number of iterations and number of simulation iterations were adjusted to 2.5, 10, 20000, and 50000, resp.) [17].

We evaluated the methodological and clinical heterogeneity of the included studies and compared the fitting degrees of the random and fixed effects models. If there was statistical homogeneity (*I*^2^ ≤ 50%, *P* ≥ 0.1) in the subgroup, the fixed effects model was adopted for NMA. If no statistical heterogeneity (*I*^2^ > 50%, *P* < 0.1) was found, the random effects model was employed for NMA, and the potential causes of heterogeneity were determined based on methodological and clinical aspects. Descriptive analyses were performed when the RCT data could not be meta-analyzed.

The point split model was utilized to examine for inconsistency. If no statistical difference (*P* > 0.05) was observed among the studies in the subgroup, the consistency model was adopted for NMA; otherwise, the inconsistent model was applied. Convergence efficiency was tested by the potential scale reduced factor (PSRF). The results of consistency model analysis were considered reliable when a good convergence efficiency was achieved (PSRF = ∼1).

## 3. Results

### 3.1. Results for Literature Searching

There were 547 studies during initial searching, and 52 clinical control studies were ultimately included after step-by-step screening.  Figure 1 shows the procedure and data for literature screening.

### 3.2. Baseline Information and Quality Evaluation of Inclusion Studies

**Figure 2:**
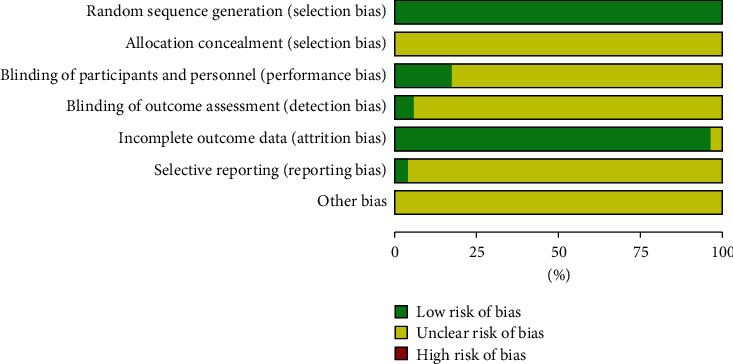
Bias risk assessment for inclusion in the study.

Fifty-two RCTs with 4711 patients with PSD were included. The experimental group included 18 Wuling capsules, 16 Shugan Jieyu capsules, 6 Yangxue Qingnao granules, 5 Danzhi Xiaoyao pills, 4 Xiaoyao pills, 2 Jieyu Anshen capsules, and 1 Chaihu Shugan powder combined with western medicines. Studies in the control groups all involved western medicines. The dose and method of administration of western medicines in both groups were the same.  Table 1 shows the baseline information of the included RCTs [18–69]. Fifty-two RCTs were double arm trials and all of them mentioned randomized grouping, and nine mentioned the instructions for a blind method and did not mention the concealment of random allocation, selective reporting, and other biases (see Figure 2 for quality evaluation).

### 3.3. Consistency Analysis and Network Diagram

**Figure 3:**
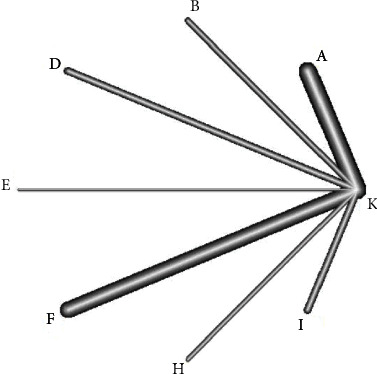
Evidence network diagram of CPMs in the auxiliary treatment of PSD. All abbreviations of CPM represent the combination group of CPM combined with western medicine, rather than CPM alone (code A: Wuling capsule combined with western medicine, B: Xiaoyao pill combined with western drugs, D: Danzhi Xiaoyao pill combined with western drugs, E: Chaihu Shugan powder combined with western drugs, F: Shugan Jieyu capsule combined with western medicine, H: Jieyu Anshen capsule combined with western medicine, I: Yangxue Qingnao granule combined with western medicine. K: blank control; this blank control refers to the type of CPM combined with western drug/positive control drug applied in the study, that is, the dosage and method of administration of the two groups of western medicine/positive control drug are consistent, so it belongs to the blank control trial design).

Figure 3 shows the included two-arm studies. The consistency analysis of four outcome indicators, that is, the clinical total effective rate, HAMD score, NIHSS score, and TESS score, was conducted. The PSFR value of the parameter was 1.00, indicating that the data results had good convergence, so the NMA was conducted under the consistency model. The network relationship was between interventions in the treatment of PSD. The numbers in the figure indicate the number of randomized controlled studies that were directly compared. The solid line in the figure indicates that there was a direct comparison between the two interventions, and the unconnected line indicates that the original study has not been directly compared. RCT can compare indirect relationships through network meta-analysis.

### 3.4. NMA Results

#### 3.4.1. Effective Rate

The total effective rate is the OR, as the effect size.  Table 2 shows that the intervention measures were compared with blank controls. Wuling capsule [OR = 4.35, 95% CI (3.03–6.26)], Xiaoyao pill [OR = 4.05, 95% CI (1.98–9.03)], Danzhi Xiaoyao pill [OR = 3.81, 95% CI (2.09–7.03)], Chaihu Shugan powder [OR = 3.12, 95% CI (0.57–8.97)], Shugan Jieyu capsule [OR = 3.87, 95% CI (2.77–6.05)], Jieyu Anshen capsule [OR = 5.00, 95% CI (1.72–9.48)], and Yangxue Qingnao granule [OR = 2.47, 95% CI (1.50–4.27)] showed clinical efficacy, and the differences were statistically significant. A pairwise comparison of seven interventions found that the Wuling capsule was better than Danzhi Xiaoyao pills [OR = 1.14, 95% CI (0.56–2.32)], Shugan Jieyu capsules [OR = 1.11, 95% CI (0.68–1.86)], or Yangxue Qingnao granules [OR = 1.76, 95% CI (0.93–3.26)] adjuvant therapy. Xiaoyao pills were better than Danzhi Xiaoyao pills [OR = 1.08, 95% CI (0.38–2.91)], Shugan Jieyu capsules [OR = 1.05, 95% CI (0.47–2.45)], or Yangxue Qingnao granules [OR = 1.63, 95% CI (0.69–4.15)] adjuvant therapy. The Danzhi Xiaoyao pill was better than the Shugan Jieyu capsule [OR = 0.98, 95% CI (0.49–2.02)] or Yangxue Qingnao granules [OR = 1.55, 95% CI (0.70–3.36)] adjuvant therapy. Chaihu Shugan powder was better than Shugan Jieyu capsules [OR = 0.81, 95% CI (0.14–8.57)] or Yangxue Qingnao granules [OR = 1.29, 95% CI (0.21–13.68)] adjuvant therapy. The Shugan Jieyu capsule was better than Yangxue Qingnao granules [OR = 1.56, 95% CI (0.84–3.18)]. The Jieyu Anshen capsule was better than Yangxue Qingnao granules [OR = 2.03, 95% CI (0.60–8.83)] as adjuvant therapy, and there was no obvious difference in other pairwise comparisons, as shown in Table 2.

**Figure 4:**
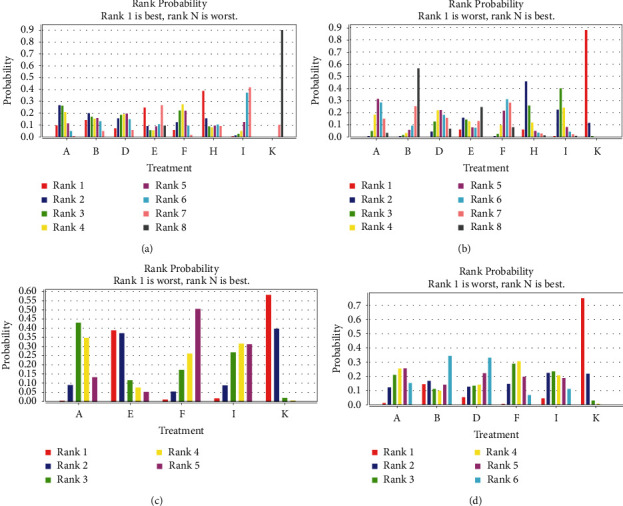
Ranking diagram of different outcome indicators for each intervention: (a) total effective rate; (b) HAMD score; (c) NIHSS score; (d) TESS total score. The higher the total effective rate (Rank 1), the greater the effects. The smaller the HAMD, NIHSS, and TESS (Rank 1), the greater the effects.

Probability ranking was as follows: Jieyu Anshen capsule (0.39) > Chaihu Shugan powder (0.25) > Xiaoyao pill (0.14) > Wuling capsule (0.10) > Danzhi Xiaoyao Pill (0.07) > Shugan Jieyu capsule (0.06) > Yangxue Qingnao granule (0.00). See Table 3 and Figure 4(a) for details.

#### 3.4.2. HAMD Score

Forty-eight studies reported a comparison of the relevant Hamilton Depression Scale scores, and the network relationship between the comparisons of various interventions is shown in Figure 4(b). Taking MD as the effect quantity, the 95% CI confidence interval was used for analysis and statistics.  Table 2 shows that each of the following was compared with the blank control and Wuling capsule [MD = −3.95, 95% CI(−4.88–−3.00)], Xiaoyao pills [MD = −5.19, 95% CI (−7.07–3.27)], Danzhi Xiaoyao pills [MD = −3.78, 95% CI(−5.55–1.84)], Shugan Jieyu capsules [MD = −4.22, 95% CI(−5.23–3.17)], and Yangxue Qingnao granules [MD = −2.65, 95% CI(−4.37–0.97)], and all were statistically significant, and no obvious difference was found between other pairwise comparisons (*P* > 0.05).

Probability rankings were as follows: Wuling capsule (0.00) = Shugan Jieyu capsule (0.00) = Yangxue Qingnao granule (0.00) = Xiaoyao pill (0.00) = Danzhi Xiaoyao pill (0.00) > Chaihu Shugan powder (0.05) > Jieyu Anshen capsule (0.06), as shown in Table 3 and Figure 4(b).

#### 3.4.3. NIHSS Score

Eleven studies reported NIHSS score analysis. OR was the effect measure.  Table 2 shows that each intervention was compared with the blank control. Wuling capsule [OR = −3.25, 95% CI (−5.46–1.05)] and Shugan Jieyu capsule [OR = −4.37, 95% CI (−8.19–0.57)] ATs have been shown to be effective in reducing NIHSS scale scores, and the differences were statistically significant. No remarkable difference was found between the two intervention measures (*P* > 0.05), as shown in Table 2.

Probability rankings were as follows: Wuling capsule (0.00) > Shugan Jieyu capsule (0.01) > Yangxue Qingnao granule (0.02) > Chaihu Shugan powder (0.39). See Table 3 and Figure 4(c) for details.

#### 3.4.4. TESS Score

Fourteen studies reported adverse reaction analysis. OR was the effect quantity.  Table 2 shows that each intervention was compared with the blank control. Wuling capsule [OR = 0.22, 95% CI (0.05–0.79)], Xiaoyao pill [OR = 0.21, 95% CI (0.01–4.14)], Danzhi Xiaoyao Pill [OR = 0.17, 95% CI (0.01–1.78)], Shugan Jieyu capsule [OR = 0.26, 95% CI (0.10–0.58)], and Yangxue Qingnao granules [OR = 0.28, 95% CI (0.05–1.42)] ATs have shown to be effective in reducing the incidence of clinical adverse reactions, and the differences were statistically significant. There was no remarkable difference in other pairwise comparisons (*P* > 0.05). See Table 2.

Probability rankings were as follows: Wuling capsule (0.00) > Shugan Jieyu capsule (0.01) > Yangxue Qingnao granule (0.04) > Danzhi Xiaoyao Pill (0.05) > Xiaoyao pill (0.14). See Table 3 and Figure 4(d).

## 4. Discussion

TCM suggests that PSD belongs to the combined category of “stroke” and “depression syndrome.” We should reasonably apply the integrated regulation and individual syndrome differentiation and treatment methods of TCM and use products that sooth the liver and relieve the depression, strengthening the spleen and stomach, alleviating depression to regulate qi, nourishing yin and promoting body fluids, and supplementing qi and strengthening the stomach. In this way, it can not only balance qi, blood, yin and yang, and dredging meridian and relax emotions but also effectively exert the clinical advantages of pure Chinese medicine preparation.

TCM is usually applied for promoting qi, soothing the liver, dredging collaterals, relieving depression, nourishing yin, activating blood, and removing blood stasis. It is not only a multitarget herb but also effectively induces the synergy of effective antidepressive components. In this NMA, seven types of oral CPMs were screened by data mining, including Wuling capsule, Shugan Jieyu capsule, Jieyu Anshen capsule, Yangxue Qingnao granule, Chaihu Shugan powder, Danzhi Xiaoyao pill, and Xiaoyao pill, which were prepared by natural Chinese herbal medicines. On the basis of definite curative effect, Chinese herbal medicine can decrease the occurrence of side effects and greatly improve medication adherence and tolerance. With respect to dosage form, CPMs can not only prevent aggravation of Chinese medicine decoction but also enhance the flavor of CPMs through sucrose-based auxiliary materials, which are preferred by patients clinically. Hence, CPMs have potential clinical applications in PSD [70, 71].

The network meta-analysis concluded that the first ranking for clinical effectiveness was the Jieyu Anshen capsule adjuvant therapy, the second was Chaihu Shugan powder, and the third was the Xiaoyao pill. Through these three proprietary Chinese medicine formulas, it can be found that all have good effects of relieving depression and soothing the liver, invigorating the spleen and regulating qi, nourishing blood, and calming the nerves. Among them, the Jieyu Anshen capsule was also added with Rhizoma *Acorus gramineus*, *Polygala tenuifolia*, Curcumae Radix, Pinellia, and other important products for calming the nerves, resolving phlegm. Modern studies have found that the water extract of *Acorus tatarinowii* has a certain antidepressant effect, which may be related to the improvement of 5-HT nerve function in the brain. At the same time, compatibility with saikosaponin can effectively enhance the ability to inhibit 5-HT reuptake to enhance the antidepressant effect. The combination of Polygala, Dan Nan Xing, and Pinellia ternata can enhance the effects of relieving depression, calming nerves, exempting phlegm, and resuscitation, which coincides with the syndrome of depression caused by stroke. Therefore, the combination of various medicines not only takes into account the pathogenic factors of qi and blood stagnation caused by apoplexy but also pays attention to the pathogenic characteristics of the depression syndrome, so it can accelerate the repair of nerve cells and nerve functions and has a high clinical efficacy [72, 73]. Chaihu Shugan powder and Xiaoyao pills are evolved from the classical famous prescription Sini powder, and both contain products for soothing the liver and relieving depression, nourishing the blood, nourishing the liver, and softening the liver. At the same time, they are combined with herbals for regulating qi and activating blood circulation, which can effectively disperse the stagnation of liver qi and stasis in the circulation of blood, so as to make liver qi smooth, blood deficiency supplement, and strengthen spleen. Pharmacological studies have confirmed that the two prescriptions not only regulate the cerebral cortex and improve cerebral microcirculation but also regulate immune and antioxidant functions, so they can promote the rehabilitation of patients with PSD.

Regarding the HAMD, NIHSS, and TESS scale scores, the AT of Wuling capsules can effectively reduce the relevant scale factor scores. This medicinal preparation is a dry powder of mycelium produced by fermentation of strains isolated from Wuling Shen. It contains a variety of adenosine, polysaccharides, amino acids, vitamins, and trace elements, which can have antianxiety and antidepressant effects and has two-way regulation of cerebral cortex function. It can inhibit the synthesis of the neurotransmitter *γ*-aminobutyric acid and improve the binding of related receptors in cerebral cortex. Moreover, it can enhance the brain energy reserves to protect the damaged brain nerve cells, so as to promote the healing force of nerve function defects, nerve cell repair force, calming, and tranquilizing force and has a better force in reducing the toxic and side effects of drugs. Therefore, it is considered that the auxiliary treatment of CPM has certain clinical value, safety, and reliability [74].

Through the comprehensive data analysis, the most effective CPM was selected to provide certain reference values for clinicians. There are some limitations to this study, including selection bias, clinical heterogeneity, and publication bias, which may influence the study outcomes. However, we suggest that this NMA can provide reliable reference value for clinical practice and evidence-based medicine as well as selecting the most appropriate treatment option for PSD to a certain extent. The protocol for this NMA has been registered on the international system review expectation register (CRD42020164543), which follows the guidelines of “Cochrane Intervention System Review Manual” and “PRISMA-P statement.” There will be a description of the amendment with date and reason if the protocol needs to be amended.

## 5. Conclusion

In summary, the Jieyu Anshen capsule is the first choice to improve clinical efficacy in the treatment of PSD. To reduce the HAMD and NIHSS scale scores and improve security, Wuling capsules should be the first choice for adjuvant treatment for the best effect. However, the specific disease should also be combined with the actual situation of patients and syndrome differentiation to make a reasonable choice for medication. Through extensive collection of the literature, related combination, and statistic analysis, this study provided reference value for clinicians when optimizing the choice of proprietary CPM for adjuvant therapy. However, the study is included in the single center clinical randomized control trial, and the number of samples included was relatively small, which made the statistical efficiency low and affected the stability of long-term efficacy evaluation results. Therefore, the design of future trials should be verified by large-scale, multicenter, prospective, double-blind randomized controlled trials, and the objective criteria should be used to evaluate the indicators, so as to reduce the risk of personal bias to the greatest extent, and provide a reliable basis for evaluation of results that can effectively guide the clinical selection of prescriptions and drugs.

## Figures and Tables

**Table 1:** Basic information included in the study.

Included study	Cases (T/C)	Age	Intervention measures		Course of treatment (d)	Outcome indicators
			Test group (T)	Control group (C)		
Han xuqing 2014 [18]	28/30	59.29 ± 10.4	Wuling capsules 2 capsules, bid + Citalopram 10 mg/d, qd	Citalopram 10 mg/d, qd	24	①②
Wu yuhong 2013[19]	35/35	55∼77	Wuling capsules 3 capsules, tid + Dealixin 1 tablet, bid	Dealixin 1 tablet, bid	56	①②
Guo ying 2013[20]	95/95	68.14 ± 7.62	Wuling capsules 3 capsules, tid + Dealixin 1 tablet, bid	Dealixin 1 tablet, bid	42	①②③④
Mo shaozhen 2015[21]	69/69	45∼75	Wuling capsules 3 capsules, tid + Dealixin 1 tablet, tid	Dealixin 1 tablet, tid	42	①②
Ma yunzhi 2012 [22]	30/30	64.8 ± 9.3	Wuling capsules 3 capsules, tid + Dealixin 1 tablet, tid	Dealixin 1 tablet, tid	42	①②③④
Zhou hongye 2015 [23]	34/32	47∼74	Wuling capsules 3 capsules, tid + Dealixin 1 tablet, tid	Dealixin 1 tablet, tid	84	①⑤
Chen liang 2015 [24]	34/34	46∼75	Wuling capsules 3 capsules, tid + Dealixin 1 tablet, tid	Dealixin 1 tablet, tid	42	①②
Zhang lili 2010 [25]	45/45	45∼74	Wuling capsules 3 capsules, tid + Dealixin 1 tablet, tid	Dealixin 1 tablet, tid	42	①②
Liang yuan 2014 [26]	30/30	55.12 ± 5.2	Wuling capsules 3 capsules, tid + Dealixin 1 tablet, tid	Dealixin 1 tablet, tid	42	①②
Zhang danni 2016 [27]	50/50	52∼77	Wuling capsules 3 capsules, tid + Fluoxetine 20 mg/d, qd	Fluoxetine 20 mg/d, qd	42	①②
Ma binfeng 2017 [28]	63/63	58∼86	Wuling capsules 3 capsules, tid + Fluoxetine 20 mg/d, qd	Fluoxetine 20 mg/d, qd	56	①②
Shi qi 2008 [29]	30/26	46∼82	Wuling capsules 3 capsules, tid + Fluoxetine 20 mg/d, qd	Fluoxetine 20 mg/d, qd	42	①②
Kou jianhua 2019 [30]	40/40	42∼75	Wuling capsules 3 capsules, tid + Fluoxetine 20 mg/d, qd	Fluoxetine 20 mg/d, qd	84	①②④
Kong xiangfang 2014 [31]	38/38	43∼76	Wuling capsules 3 capsules, tid + Fluoxetine 20 mg/d, qd	Fluoxetine 20 mg/d, qd	56	①②④
Yuan jun 2014 [32]	85/85	45∼75	Wuling capsules 3 capsules, tid + Paroxetine 20 mg/d, qd	Paroxetine 20 mg/d, qd	28	①②④
Wan ailan 2006 [33]	35/35	59.23 ± 8.3	Wuling capsules 3 capsules, tid + Paroxetine 20 mg/d, qd	Paroxetine 20 mg/d, qd	42	①②
Liu junqiong 2014 [34]	32/32	52∼78	Wuling capsules 3 capsules, tid + Sertraline 50 mg/d, qd	Sertraline 50 mg/d, qd	56	①②
Xie yan 2018 [35]	49/49	52∼78	Wuling capsules 3 capsules, tid + Sertraline 50 mg/d, qd	Sertraline 50 mg/d, qd	56	①②③④
Zhou peng 2017 [36]	34/34	18∼68	Xiaoyao pill 8 pills, tid + Venlafaxine 75 mg/d, qd	Venlafaxine 75 mg/d, qd	28	①②
Wang jianqiang 2014 [37]	60/52	45∼75	Xiaoyao pill 8 pills, tid + Deanxit 1 tablet, bid	Deanxit 1 tablet, bid	42	①②③
Zou lihua 2009 [38]	30/30	67.9 ± 6.1	Xiaoyao pill 8 pills, tid + Deanxit 1 tablet, bid	Deanxit 1 tablet, bid	42	①②
Ceng miaolin 2018 [39]	43/43	33∼79	Xiaoyao pill 8 pills, tid + Fluoxetine 20 mg/d, qd	Fluoxetine 20 mg/d, qd	28	①②
Jiang limin 2019 [40]	74/74	36∼76	Danzhi Xiaoyao powder 3 g/d, bid + Citalopram 10 mg/d, qd	Citalopram 10 mg/d, qd	28	①②
Xu erping 2006 [41]	35/35	55.2 ± 1.9	Danzhi Xiaoyao powder 6 g/d, bid + Fluoxetine 20 mg/d, qd	Fluoxetine 20 mg/d, qd	56	①②
Wang zongyuan 2008 [42]	36/36	55∼79	Danzhi Xiaoyao powder 6 g/d, bid + Fluoxetine 20 mg/d, qd	Fluoxetine 20 mg/d, qd	84	①②
Zhang yumao 2014 [43]	40/40	47∼73	Danzhi Xiaoyao powder 6 g/d, bid + Sertraline 50 mg/d, qd	Sertraline 50 mg/d, qd	56	①②③
Peng xianwen 2014 [44]	49/49	45∼78	Danzhi Xiaoyao powder 6 g/d, bid + Fluoxetine 20 mg/d, qd	Fluoxetine 20 mg/d, qd	28	①②
Cui yi 2016 [45]	30/30	45∼80	Chaihu Shugan powder granules + citalopram 10 mg/d, qd	Citalopram 10 mg/d, qd	42	①②④
Wen jun 2015 [46]	60/60	50∼83	Shugan Jieyu capsule 2 capsules, bid + Mirtazapine 30 mg/d, qd	Mirtazapine 30 mg/d, qd	42	①②③④
Chen aijun 2013 [47]	39/39	49∼78	Shugan Jieyu capsule 2 capsules, bid + Paroxetine 20 mg/d, qd	Paroxetine 20 mg/d, qd	42	①③
Hou jihong 2015 [48]	36/36	69.3 ± 7.5	Shugan Jieyu capsule 2 capsules, bid + Paroxetine 20 mg/d, qd	Paroxetine 20 mg/d, qd	56	①②
Wu hongyi 2015 [49]	40/40	50∼65	Shugan Jieyu capsule 2 capsules, bid + Deanxit 1 tablet, qd	Deanxit 1 tablet, qd	28	①②
Zhao zheng 2013 [50]	40/40	43∼75	Shugan Jieyu capsule 2 capsules, bid + Paroxetine 20 mg/d, qd	Paroxetine 20 mg/d, qd	42	①②
Ding na 2014 [51]	40/40	56.42 ± 5.18	Shugan Jieyu capsule 2 capsules, bid + Paroxetine 20 mg/d, qd	Paroxetine 20 mg/d, qd	56	①②
Na wanqiu 2012 [52]	41/39	71.12 ± 5.51	Shugan Jieyu capsule 2 capsules, bid + Sertraline 50 mg/d, qd	Sertraline 50 mg/d, qd	56	①②③
Hu jun 2013 [53]	45/44	56.42 ± 5.18	Shugan Jieyu capsule 2 capsules, bid + Sertraline 50 mg/d, qd	Sertraline 50 mg/d, qd	42	①②
Tan hongyang 2018 [54]	62/62	52∼73	Shugan Jieyu capsule 2 capsules, bid + Paroxetine 20 mg/d, qd	Paroxetine 20 mg/d, qd	21	①②③④
Lu yi 2015 [55]	65/65	45∼72	Shugan Jieyu capsule 2 capsules, bid + Olanzapine 2.5 mg/d, qd	Olanzapine 2.5 mg/d, qd	56	①②
Xu ming 2012 [56]	65/65	55∼74	Shugan Jieyu capsule 2 capsules, bid + Venlafaxine 75 mg/d, bid	Venlafaxine 75 mg/d, bid	42	①②
Liu wei 2016 [57]	38/38	50∼75	Shugan Jieyu capsule 2 capsule, bid + Paroxetine 20 mg/d, qd	Paroxetine 20 mg/d, qd	56	①②③
Chen wei 2014 [58]	58/57	45∼73	Shugan Jieyu capsule 2 capsules, bid + Venlafaxine 75 mg/d, bid	Venlafaxine 75 mg/d, bid	42	①③
Yi kunchang 2018 [59]	48/48	48∼77	Shugan Jieyu capsule 2 capsules, bid + Paroxetine 20 mg/d, qd	Paroxetine 20 mg/d, qd	56	①②
Pan zhenshan 2014 [60]	42/42	65.12 ± 8.35	Shugan Jieyu capsule 2 capsules, bid + Mirtazapine 30 mg/d, qd	Mirtazapine 30 mg/d, qd	56	①②③
Li junling 2013 [61]	27/27	49∼75	Shugan Jieyu capsule 2 capsules, bid + Fluoxetine 20 mg/d, qd	Fluoxetine 20 mg/d, qd	56	①②
Xie yan 2017 [62]	45/45	55∼70	Jieyu Anshen granules 5 g, bid + Deanxit 1 pill, qd	Deanxit 1 pill, qd	42	①②
Xia junbo 2013 [63]	40/40	34∼72	Jieyu Anshen granules 5 g, bid + Fluoxetine 20 mg/d, qd	Fluoxetine 20 mg/d, qd	56	①②
Mu ying 2014 [64]	48/48	40∼78	Yangxue Qingnao granules 4 g/d, tid + Paroxetine 20 mg/d, qd	Paroxetine 20 mg/d, qd	28	①④
Jiang guohua 2018 [65]	60/60	60∼77	Yangxue Qingnao granules 4 g/d, tid + Fluoxetine 20 mg/d, qd	Fluoxetine 20 mg/d, qd	56	①②③④
Huang xiaohong 2012 [66]	50/50	61.5 ± 7.8	Yangxue Qingnao granules 4 g/d, tid + Sertraline 50 mg/d, qd	Sertraline 50 mg/d, qd	56	①②
Pan dong 2014 [67]	38/41	60∼75	Yangxue Qingnao granules 4 g/d, tid + Sertraline 50 mg/d, qd	Sertraline 50 mg/d, qd	56	①②
Ceng zhaofu 2013 [68]	34/34	41∼75	Yangxue Qingnao granules 4 g/d, tid + Fluoxetine 20 mg/d, qd	Fluoxetine 20 mg/d, qd	56	①②
Wang xuejun 2017 [69]	32/32	61.37 ± 6.26	Yangxue Qingnao granules 4 g/d, tid + Citalopram 10 mg/d, qd	Citalopram 10 mg/d, qd	42	①②③

① Total effective rate; ② HAMD score (Hamilton Depression Scale); ③ TESS total score (adverse reactions); ④ NIHSS score (neurological deficit score). The intervention measures were based on the routine treatment of stroke..

**Table 2:** Results of network meta-analysis.

Therapeutic method		Clinical effective rate	HAMD score	NIHSS score	TESS score
Method 1	Method 2				
Wuling capsule	Xiaoyao pill	1.08[0.44,2.38]	1.24[−0.88,3.38]	—	1.05[0.04,6.21]
	Danzhi Xiaoyao pill	1.14[0.56,2.32]^*∗*^	−0.17[−2.30,1.80]	—	1.31[0.08,2.00]
	Chaihu Shugan powder	1.40[0.13,8.00]	−0.28[−5.07,4.61]	−2.62[−8.57,3.19]	—
	Shugan Jieyu capsule	1.11[0.68,1.86]^*∗*^	0.26[−1.09,1.64]	1.10[−3.31,5.56]	0.84[0.16,4.36]
	Jieyu Anshen capsule	0.87[0.22,2.88]	−1.94[−4.66,0.90]	—	—
	Yangxue Qingnao granule	1.76[0.93,3.26]^*∗*^	−1.29[−3.24,0.66]	0.50[−3.86,4.86]	0.78[0.08,6.23]
	Blank control	4.35[3.03,6.26]^*∗*^	−3.95[−4.88,−3.00]^*∗*^	−3.25[−5.46,−1.05]^*∗*^	0.22[0.05,0.79]^*∗*^

Xiaoyao pill	Danzhi Xiaoyao pill	1.08[0.38,2.91]^*∗*^	−1.40[−4.18,1.15]	—	1.23[0.01,6.62]
	Chaihu Shugan powder	1.27[0.11,8.52]	−1.50[−6.64,3.65]	—	—
	Shugan Jieyu capsule	1.05[0.47,2.45]^*∗*^	−0.97[−3.14,1.20]	—	0.80[0.02,9.13]
	Jieyu Anshen capsule	0.82[0.16,3.07]	−3.18[−6.42,0.07]	—	—
	Yangxue Qingnao granule	1.63[0.69,4.15]^*∗*^	−2.54[−5.08,0.04]	—	0.73[0.01,2.65]
	Blank control	4.05[1.98,9.03]^*∗*^	−5.19[−7.07,−3.27]^*∗*^	—	0.21[0.01,4.14]^*∗*^

Danzhi Xiaoyao pill	Chaihu Shugan powder	1.21[0.12,7.34]	−0.10[−5.11,5.03]	—	—
	Shugan Jieyu capsule	0.98[0.49,2.02]^*∗*^	0.43[−1.59,2.61]	—	0.65[0.05,8.75]
	Jieyu Anshen capsule	0.74[0.16,2.61]	−1.77[−4.82,1.60]	—	—
	Yangxue Qingnao granule	1.55[0.70,3.36]^*∗*^	−1.13[−3.57,1.48]	—	0.59[0.03,11.33]
	Blank control	3.81[2.09,7.03]^*∗*^	−3.78[−5.55,−1.84]^*∗*^	—	0.17[0.01,1.78]^*∗*^

Chaihu Shugan powder	Shugan Jieyu capsule	0.81[0.14,8.57]^*∗*^	0.55[−4.31,5.35]	3.72[−2.84,10.49]	—
	Jieyu Anshen capsule	0.62[0.07,9.94]	−1.62[−7.06,3.70]	—	—
	Yangxue Qingnao granule	1.29[0.21,13.68]^*∗*^	−1.03[−6.04,4.03]	3.14[−3.39,9.76]	—
	Blank control	3.12[0.57,8.97]^*∗*^	−3.65[−8.45,1.05]	−0.63[−5.99,4.87]	—

Shugan Jieyu capsule	Jieyu Anshen capsule	0.77[0.18,2.88]	−2.20[−5.00,0.66]	—	—
	Yangxue Qingnao granule	1.56[0.84,3.18]^*∗*^	−1.57[−3.55,0.46]	−0.60[−6.06,4.86]	0.93[0.14,5.67]^*∗*^
	Blank control	3.87[2.77,6.05]^*∗*^	−4.22[−5.23,−3.17]^*∗*^	−4.37[−8.19,−0.57]^*∗*^	0.26[0.10,0.58]^*∗*^

Jieyu Anshen capsule	Yangxue Qingnao granule	2.03[0.60,8.83]^*∗*^	0.65[−2.44,3.76]	—	—
	Blank control	5.00[1.72,9.48]^*∗*^	−2.00[−4.67,0.56]	—	—

Yangxue Qingnao granule	Blank control	2.47[1.50,4.27]^*∗*^	−2.65[−4.37,−0.97]^*∗*^	−3.76[−7.56,0.03]	0.28[0.05,1.42]^*∗*^

**Table 3:** Ranking list of different interventions.

Intervention measures	Clinical effective rate	HAMD score	NIHSS score	TESS score
Wuling capsule	0.10	0.00	0.00	0.00
Xiaoyao pill	0.14	0.00	0.14	—
Danzhi Xiaoyao pill	0.07	0.00	0.05	—
Chaihu Shugan powder	0.25	0.05	—	0.39
Shugan Jieyu capsule	0.06	0.00	0.01	0.01
Jieyu Anshen capsule	0.39	0.06	—	—
Yangxue Qingnao granule	0.00	0.00	0.04	0.02
Blank control	0.00	0.88	0.75	0.58

## Data Availability

The data that support the findings of this study are publicly available and can be obtained from the corresponding author upon reasonable request.

## Abbreviations

HAMD: Hamilton Depression Scale
TESS: Treatment Emergent Symptom Scale
NIHSS: National Institute of Health Stroke Scale
PRISMA-P: Preferred reporting items for systematic reviews and meta-analyses protocols
NMA: Network meta-analysis
RCTS: Randomized controlled trials
ADDIS: The Aggregate Data Drug Information System
MCMC: Markov China Monte Carlo
CI: Confidence interval
OR: Odds ratio
RR: Risk ratio
MD: Mean difference
PSRF: Potential scale reduced factor
PICOS: Participants, interventions, comparators, outcomes, study design.
TCM: Traditional Chinese medicine.

## References

[1] Huff W., Steckel R., Sitzer M. (2003). Poststroke depression. *Nervenarzt, Der*.

[2] Sivolap Y. P., Damulin I. V. (2019). Stroke and depression. *Zhurnal Nevrologii i Psikhiatrii im. S.S. Korsakova*.

[3] Paolucci S. (2008). Epidemiology and treatment of post-stroke depression. *Neuropsychiatric Disease and Treatment*.

[4] Loubinoux I., Kronenberg G., Endres M. (2012). Post-stroke depression: mechanisms, translation and therapy. *Journal of Cellular and Molecular Medicine*.

[5] Shelton R. C., Osuntokun O., Heinloth A. N., Corya S. A. (2010). Therapeutic options for treatment-resistant depression. *CNS Drugs*.

[6] Yu Y., Zhang G., Liu J., Han T, Huang H (2020). Network meta-analysis of Chinese patent medicine adjuvant treatment of poststroke depression. *Medicine*.

[7] Shamseer L., Moher D., Mike C. (2015). Preferred reporting items for systematic review and meta-analysis protocols (PRISMA-P) 2015: elaboration and explanation. *BMJ Case Reports*.

[8] Neurology Branch of Chinese Medical Association, Cerebrovascular Disease Group of Neurology Branch of Chinese Medical Association (2015). Guidelines for the diagnosis and treatment of acute ischemic stroke 2014. *Chinese Journal of Neurology*.

[9] Chinese Medical Association Psychological Science Society (2001). Classification and diagnostic criteria of mental disorders in China (Third Edition). *Chinese Journal of psychiatry*.

[10] State Administration of Traditional Chinese Medicine (2012). *Diagnostic Efficacy Standard of Traditional Chinese Medicine Disease*.

[11] Hutton B., Salanti G., Caldwell D. M. (2015). The PRISMA extension statement for reporting of systematic reviews incorporating network meta-analyses of health care interventions: checklist and explanations. *Annals of Internal Medicine*.

[12] Liberati A., Altman D. G., Tetzlaff J. (2009). The PRISMA statement for reporting systematic reviews and meta-analyses of studies that evaluate health care interventions: explanation and elaboration. *PLoS Medicine*.

[13] Hongqiu G., Yang W., Wei L. (2014). Application of Cochrane bias risk assessment tool in meta-analysis of randomized controlled study. *Chinese Circulation Journal*.

[14] Jie M., Ying L., Laiping Z., Chenping Z., Zhiyuan Z. (2012). Application and comparison of Jadad scale and Cochrane bias risk assessment tool in quality evaluation of randomized controlled trials. *China Journal of Oral and Maxillofacial Surgery*.

[15] Guyatt G., Oxman A. D., Akl E. A. (2011). GRADE guidelines: 1. Introduction-GRADE evidence profiles and summary of findings tables. *Journal of Clinical Epidemiology*.

[16] Chao Z., Feng S., Xiantao Z. R. (2014). Software calls JAGS software to realize network meta-analysis. *Chinese Journal of Evidence-Based Medicine*.

[17] Van Valkenhoef G., Tervonen T., Zwinkels T., De Brock B., Hillege H. (2013). Addis: a decision support system for evidence-based medicine. *Decision Support Systems*.

[18] Han X. (2014). Clincal research in living quality of life on post-stroke depression patients and clinical efficacy observation of escitalopram oxalate plus Wuling Capsules. *Hubei University of Traditional Chinese Medicine*.

[19] Wu Y., Wang S., Jiang X. (2013). Deanxit and Wuling capsule in the treatment of post-stroke depression. *Guangming Journal of Chinese Medicine*.

[20] Guo Y., Zhou J. (2013). Clinical efficacy and safety of Deanxit combined with Wuling capsule in the treatment of post-stroke depression. *For all Health*.

[21] Mo S., Liao J., Gao Q. (2015). Clinical observation of Wuling capsule combined with Deanxit in the treatment of post-stroke depression. *China Medical Engineering*.

[22] Ma Y., Zheng T., Tian Y. (2012). Clinical study of Wuling capsule in the treatment of depression after ischemic stroke. *Journal of Traditional Chinese Medicine*.

[23] Zhou H. (2015). Effect of Wuling Capsule on post stroke depression. *New Journal of Traditional Chinese Medicine*.

[24] Chen L., Man X. (2015). Clinical efficacy and safety of Wuling capsule combined with Deanxit in the treatment of post-stroke depression. *Electronic Journal of Clinical Medicine Literature*.

[25] Zhang L., Mei J., Zhang S. (2010). Effect of Wuling capsule combined with Deanxit on post-stroke depression. *Journal of Contemporary Medicine*.

[26] Liang Y., Qian R. (2014). Wuling capsule combined with Deanxit in the treatment of 30 cases of post stroke depression. *Journal of Clinical Research of Traditional Chinese Medicine*.

[27] Zhang D. (2016). Efficacy of Wuling capsule combined with fluoxetine (Prozac) in the treatment of post-stroke depression and anxiety. *Journal of Northern Pharmacy*.

[28] Ma B., Wu J., Zhen J. (2017). Clinical observation of Wuling capsule combined with fluoxetine in the treatment of post-stroke depression. *Straits Pharmaceutical Journal*.

[29] Shi Q., Liu X., Chen Y. (2008). Efficacy of Wuling capsule combined with fluoxetine in the treatment of post-stroke depression and neurological deficit. *Journal of Chinese Patent Medicine*.

[30] Kou J. (2019). Observation of the effect of Wuling capsule combined with antidepressants in the treatment of post-stroke depression and its influence on patients’ neurological function. *Journal of Modern Diagnosis and Treatment*.

[31] Kong X., Xiao J., Wang K. (2014). Treatment of 38 cases of post-stroke depression with fluoxetine hydrochloride combined with Wuling capsule. *China Pharmaceutical*.

[32] Yuan J. (2014). Effect of wuling capsule on the prognosis of acute cerebral infarction patients with post-stroke depression. *Modern Chinese Doctor*.

[33] Wan A., Yuan Y. (2006). Comparison of the therapeutic effects of Wuling Capsule and Xylote in the treatment of post-stroke depression. *China Clinical Rehabilitation*.

[34] Liu J. (2014). Clinical observation of wuling capsule in improving sleep disorders in patients with post-stroke depression. *TCM clinical research*.

[35] Xie Y., Gao Z., Wang X. (2018). The clinical efficacy of Wuling capsule combined with western medicine in the treatment of patients with stroke and depression and its effect on serum inflammatory factors and NPY. *World Traditional Chinese Medicine*.

[36] Zhou P. (2017). Clinical observation on the treatment of 34 cases of post-stroke depression with venlafaxine capsule and xiaoyao powder. *Journal of Primary Medicine Forum*.

[37] Wang J., Ni X. (2014). Observation on the effect of flupentixol and melitrazin combined with Xiaoyao Pills in treating depression. *Practical Journal of Cardio-Cerebral Pulmonary Vascular Disease*.

[38] Zou L., Li H., Meng W. (2009). Clinical observation of delixin combined with xiaoyao pills in treating post-stroke depression. *Community Medicine Journal*.

[39] Ceng M., Chen L., Li B. (2018). The clinical efficacy of Xiaoyao Pill combined with fluoxetine in the treatment of post-stroke depression and its effect on serum serotonin level. *Zhejiang Journal of Integrated Traditional Chinese and Western Medicine*.

[40] Jiang L., Liu X. (2019). Efficacy and mechanism of Danzhi Xiaoyao San in the treatment of post-stroke depression. *Chinese Journal of Experimental Traditional Medical Formulae*.

[41] Xu E. (2006). Danzhi Xiaoyao San combined with fluoxetine in the treatment of 70 cases of post-stroke depression. *Journal of Traditional Chinese Medicine Research*.

[42] Wang Z. (2008). Observation on the efficacy of fluoxetine combined with Jiawei Xiaoyao Pills in the treatment of post-stroke depression. *Medical Forum Magazine*.

[43] Zhang Y., Guo Y., Chen X. (2014). Clinical effect of Zolofu combined with Danzhi Xiaoyao Pills in the treatment of post-stroke depression. *Guide of China Medicine*.

[44] Peng X. (2014). Clinical effect of Jiawei Xiaoyao Pills combined with fluoxetine on post-stroke depression. *Practical Clinical Journal of Integrated Traditional Chinese and Western Medicine*.

[45] Cui Y. (2016). *Clinical Observation of Jiawei Chaihu Shugan Powder Combined with Escitalopram Oxalate on Post-stroke Depression*.

[46] Wen J., Li Y., Li M. (2015). Effects of mirtazapine tablets combined with Shugan Jieyu capsule on neurological function in patients with post-stroke depression. *Hebei Medical Journal*.

[47] Chen A. (2013). Clinical observation of paroxetine combined with Shugan Jieyu capsule in the treatment of post-stroke depression. *China Practical Medicine*.

[48] Hou J. (2015). Observation on the curative effect of paroxetine combined with Shugan Jieyu capsule in the treatment of post-stroke depression. *Journal of New Chinese Medicine*.

[49] Wu H., Lin P., He X. (2015). Study on the clinical efficacy of Shugan Jieyu Capsules combined with Deanxit in the treatment of post-stroke depression. *China Practical Medicine*.

[50] Zhao Z., Zhao M., Zhang S. (2013). Shugan jieyu capsule combined with paroxetine tablets in treating 40 cases of post-stroke depression. *Traditional Chinese Medicinal Research*.

[51] Ding N. (2014). A comparative study of Shugan Jieyu capsule combined with paroxetine in the treatment of post-stroke depression. *Chinese Journal of Practical Nervous Diseases*.

[52] Na W., Li J., Chen K. (2012). A comparative study of Shugan Jieyu capsule combined with sertraline in the treatment of post-stroke depression in the elderly and its influence on neurological deficits. *Zhejiang Journal of Integrated Traditional Chinese and Western Medicine*.

[53] Hu J., Yuan L., Sheng M. (2013). Observation on the curative effect of Shugan Jieyu capsule combined with sertraline in the treatment of post-stroke depression. *China Practical Medicine*.

[54] Tan H., Wan J., Xu N. (2018). Observation on the curative effect of Shugan Jieyu capsule combined with citalopram in the treatment of post-stroke depression. *Shaanxi Journal of Traditional Chinese Medicine*.

[55] Lu Y. (2015). Clinical study of Shugan Jieyu capsule combined with low-dose olanzapine in the treatment of stroke and depression. *China Modern Doctor*.

[56] Xu M., Gu L., Liu M. (2012). Observation of curative effect of shugan jieyu combined with venlafaxine hydrochloride on post-stroke depression. *Chongqing Medicine*.

[57] Liu W. (2018). *Study on the Efficacy of Shugan Jieyu Capsule Combined with Escitalopram Oxalate in the Treatment of Post-stroke Depression*.

[58] Chen W. (2014). Observation on therapeutic effect of shugan jieyu capsule on fifty-eight cases of post-stroke depression. *The Journal of Medical Theory and Practice*.

[59] Yi K., Jiang R., Zheng W. (2018). Observation on curative effect of shugan jieyu capsule in treating depression after stroke. *Chronic Pathematology Journal*.

[60] Pan Z., Zhu M., Yang J. (2018). Low-dose mirtazapine combined with Shugan Jieyu capsule in the treatment of post-stroke depression. *Journal of Henan University of Science & Technology*.

[61] Li J., Mou K. (2013). Observation on therapeutic effect of integrated traditional Chinese and western medicine on depression after stroke. *Practical Journal of Cardiac Cerebral Pneumal and Vascular Disease*.

[62] Xie Y., Xia L., Xu Y. (2017). Clinical study of flupentixol and melitroxine combined with Jieyu Anshen granules in the treatment of post-stroke depression. *Anhui Medical and Pharmaceutical Journal*.

[63] Xia J. (2013). *Study on the Effect and Mechanism of Jieyu Anshen Granules on Patients with Post-stroke Depression*.

[64] Mu Y. (2014). Clinical efficacy of paroxetine combined with Yangxue Qingnao granule in the treatment of post-stroke depression. *Chinese Journal of Practical Nervous Diseases*.

[65] Jiang G., Pu Q., Fu Y. (2018). Efficacy of Yangxueqingnao Granules combined with fluoxetine in the treatment of elderly patients with post-stroke depression and its influence on the degree of neurological deficit. *Chinese Journal of Gerontology*.

[66] Huang X. (2012). Treatment of 50 cases of post-stroke depression with Yangxue qingnao granules and sertraline. *China Pharmaceuticals*.

[67] Pan D., Gan J. (2014). Clinical observation of Yangxue qingnao granules combined with sertraline hydrochloride in treating post-stroke depression. *China Pharmacy*.

[68] Ceng Z., Zhao M. (2013). Treatment of thirty-four cases of post-stroke depression with Yangxue qingnao granule. *Clinical Journal of Chinese Medicine*.

[69] Wang X., Chu H., Duan B. (2017). Observation on the clinical effect of integrated traditional Chinese and western medicine on depression after stroke. *Electronic Journal of Clinical Medical Literature*.

[70] Li G., Li Z. K., Yang H. J. (2021). Construction of model for multidimensional evaluation of value and risk of Chinese patent medicine. *China Journal of Chinese Materia Medica*.

[71] Zhang H. L., Jiao L. W., Liang N. (2021). Construction of multi dimension and multi criteria evaluation index system for superior Chinese patent medicines based on comprehensive evaluation method. *Journal of Basic Chinese Medicine*.

[72] Li T., Sun X., Gao J. (2012). Antidepressant effect of water extract of Acorus calamus on acquired helplessness model. *Chinese Journal of Experimental Traditional Medical Formulae*.

[73] Ji N., Li J., Li M. (2006). The effect of Bupleurum fulvum on the antidepressant effect of Shichangpu alcohol precipitation solution. *Jiangsu Medical Journal*.

[74] Liu F., Li X., Xu Y. (2018). The intervention effect and mechanism of wuling capsule on fatigue after ischemic stroke. *Chinese Journal of Gerontology*.

